# Supplementary material for: The PI3K and MAPK/p38 pathways control stress granule assembly in a hierarchical manner
Source: Life Sci Alliance. 2019 Mar 28;2(2):e201800257. doi: 10.26508/lsa.201800257 (PMC6441495; doi:10.26508/lsa.201800257)
Supplement: Supplementary file 11 [file LSA-2018-00257_Supplemental_Data8.pdf]

Supplemental Data S8

Parameter fixation approach

This document includes the principal workflow used for parameter fixation to derive the mTOR model version V (see main manuscript). In this model version, stress inputs are simulated on PI3K, Akt–pS473 and the mTORC1 readouts PRAS40–pS183, p70-S6k–pT389 and 4E-BP1–pT37/46.
In brief, the iterative workflow follows the scheme depicted in Fig 1 of repeated rounds of latin hypercube sampling (LHS) and profile likelihood estimation (PLE), which are described in detail in Raue et al. (2013). Additionally, clustering analysis is performed. Each iteration relies on fixed parameters of prior fixation rounds and finishes once all parameter values are determined.
The workflow makes use of the toolbox data2dynamics (release 170117, Raue et al. (2015)) within MATLAB version R2015b or R2016b. Next, individual steps are explained in more detail.

Abbreviations: LHS: Latin Hypercobe Sampling; PLE: Profile Likelihood Estimation

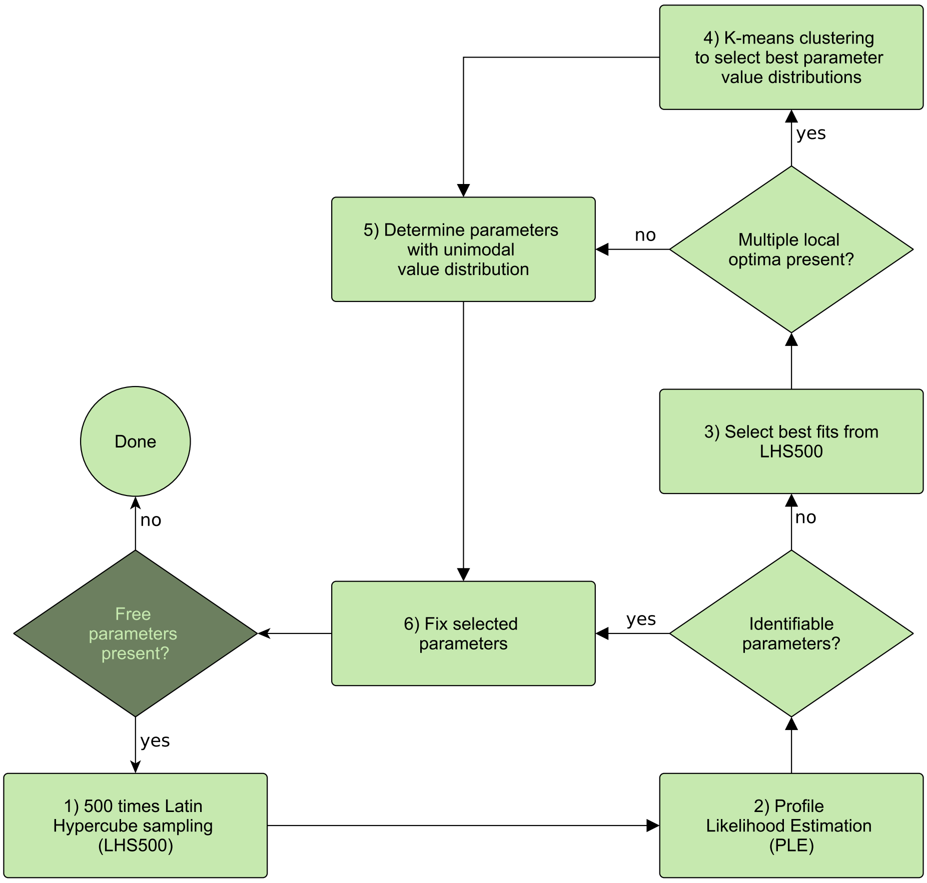

Figure 1: Scheme of parameter fixation workflow. The workflow is followed in an iterative manner as long as free (not fixed) parameters are left. Individual steps are explained in detail in the corresponding sections of this document.

1 Latin Hypercube Sampling

Initially, parameter values are sought that best fit the data by optimising the squared error (resembled by the chi^2 value) between data and model simulation.
To ensure optimality of determined parameter values the optimisation (gradient descent for local identification of optimal parameter distribution) is started multiple times with different starting values. This is achieved by using Latin hypercube sampling (LHS) that efficiently scans the high dimensional solution space (Raue et al., 2013). We used 500 repetitions (termed LHS500) as a compromise between computational demand and ensuring that the found solution is sufficiently close to the global optima. Increasing the number of runs did not provide a notable improvement of the found solution. Fig 2 shows a representative plot of chi^2 values that resemble the goodness of fit in decreasing order (lower values resemble better fits). Of note, stepwise improvements of the fit resemble different local optima.

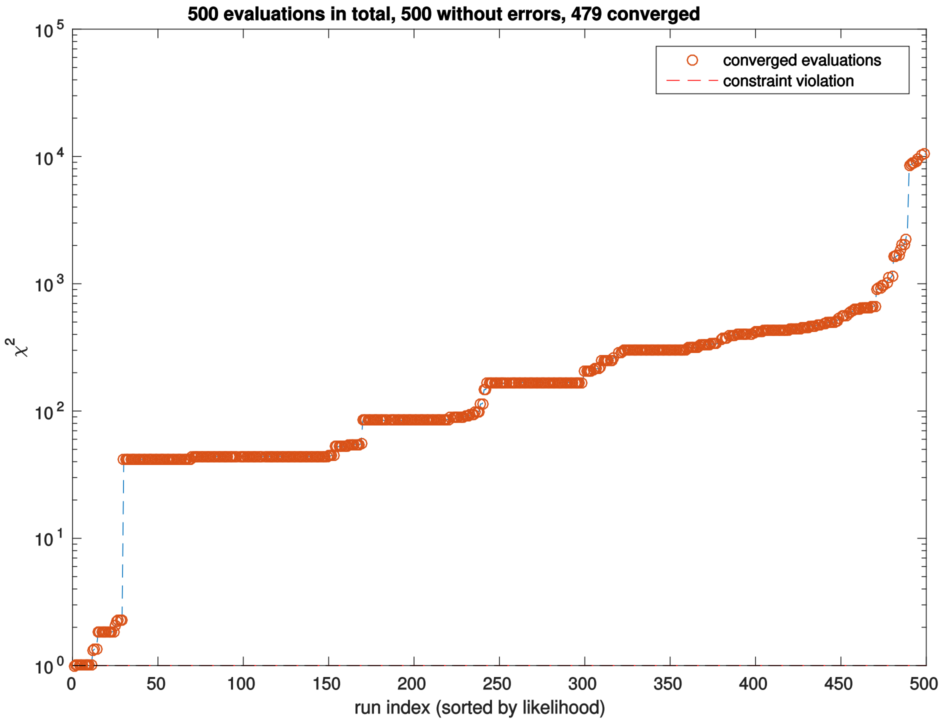

Figure 2: Result of an LHS run with 500 repetitions. The results are ordered from best to worst. Local optima are represented by steps in subsequent chi^2 values).

2 Profile Likelihood Estimation

Next, a profile likelihood estimation (PLE) is performed to determine parameters that are identifiable (Raue et al., 2010).
If there are identifiable parameters, the workflow is proceeded with step 6), otherwise with step 3) (cf. Fig 1).

3 Selecting best LHS Fits

This branch of the workflow is followed if no identifiable parameters are reported by PLE analysis. In this case LHS500 results are analysed.
Since LHS runs provide ordered goodness of fits (cf. Fig 2), in individual iterations of the workflow only the best fits are chosen for further analysis. Best fits are either chosen by selecting the best reported chi^2 values of the last LHS run before flattening out or by selecting parameter distributions corresponding to the best two local optima. If the latter is the case, the workflow is proceeded with step 4), otherwise with step 5) (cf. Fig 1).

4 K-means clustering

K-means clustering is performed for finding two clusters of parameter value distributions based on selecting the two best local optima of an LHS run. The better performing cluster in terms of mean chi^2 value is further processed in step 5). K-means clustering is repeated 100 times to provide reproducible results. Statistical significance and silhouette plots can be used to ensure fitting quality of the chosen cluster (cf. Fig 3).

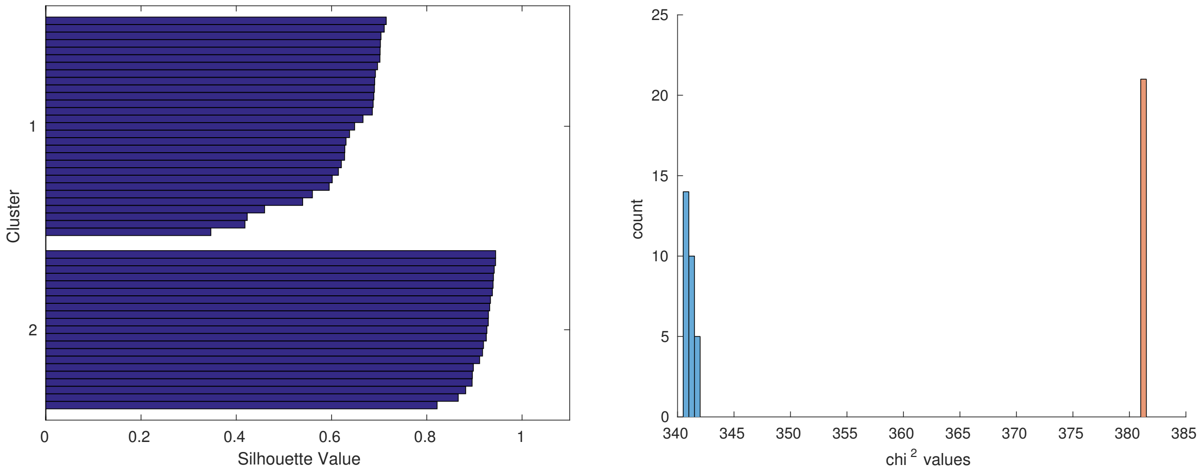

Figure 3: Results of clustering showing silhouette plot (left panel) and chi^2 value distribution of the two found cluster (right panel). As indicated by the silhouette plot the underlying parameter distributions differ notably from each other. This is confirmed by the chi^2 mean value of the preferred cluster (blue bars).

5 Identify parameters with unimodal value distribution

Next, estimated value distributions per parameter are investigated. If a clear unimodal value distributions is identified, MATLABs kernel function procedure is used to determine the value represented by the unimodal peak. All other distributions (e.g. bimodal or uniform distributions) are neglected and the respective parameter values remain free (cf. Fig 4 for representative parameter value distributions).

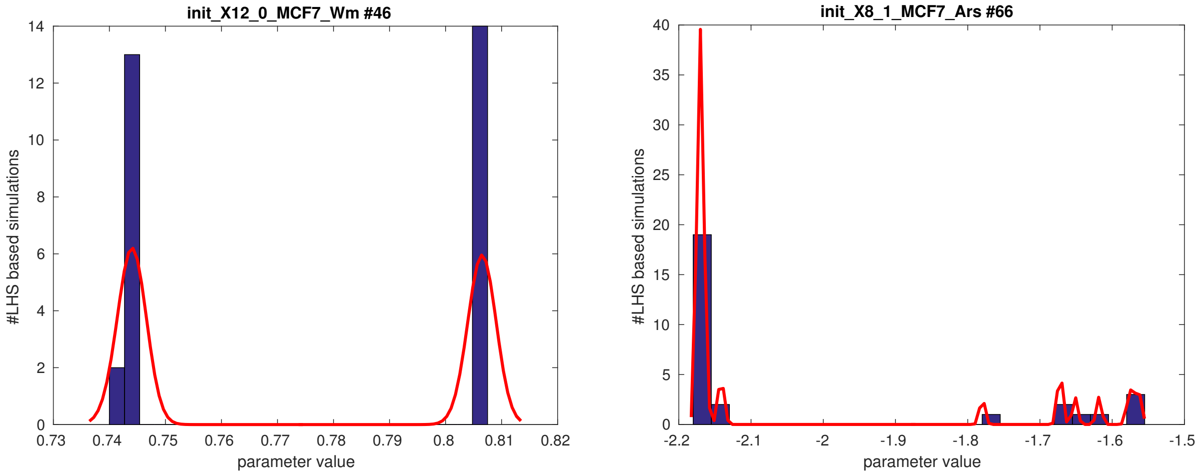

Figure 4: Two representative parameter value distributions of remaining samples from LHS run (500 times). The parameter shown in the left panel did not show a unimodal parameter value distribution and hence the parameter remained free for the respective iteration. The parameter shown in the right panel showed a sufficiently close resemblance to a unimodal distribution and hence was fixed to its highest (and only) mode.

6 Fix selected parameters

Finally, parameters that are either determined as being identifiable in step 2) or that possess a unimodal parameter distribution as determined in step 5) are determined and corresponding values are fixed for these parameters.

References

A. Raue, V. Becker, U. Klingmüller, and J. Timmer. Identifiability and observability analysis for experimental design in nonlinear dynamical models. *Chaos*, 20(4): 045105, Dec 2010. doi: 10.1063/1.3528102. URL http://dx.doi.org/10.1063/1.

A. Raue, M. Schilling, J. Bachmann, A. Matteson, M. Schelker, M. Schelke, D. Kaschek, S. Hug, C. Kreutz, B. D. Harms, F. J. Theis, U. Klingmüller, and J. Timmer. Lessons learned from quantitative dynamical modeling in systems biology. *PLoS One*, 8(9):e74335, 2013. doi: 10.1371/journal.pone.0074335. URL http://dx.doi.org/10.1371/journal.pone.0074335.

A. Raue, B. Steiert, M. Schelker, C. Kreutz, T. Maiwald, H. Hass, J. Vanlier, C. Tönsing, L. Adlung, R. Engesser, W. Mader, T. Heinemann, J. Hasenauer, M. Schilling, T. Höfer, E. Klipp, F. Theis, U. Klingmüller, B. Schöberl, and J. Timmer. Data2dynamics: a modeling environment tailored to parameter estimation in dynamical systems. *Bioinformatics*, Jul 2015. doi: 10.1093/bioinformatics/btv405. URL http://dx.doi.org/10.1093/bioinformatics/btv405.
